# Predicting the Potential Distribution of Pine Wilt Disease in China under Climate Change

**DOI:** 10.3390/insects13121147

**Published:** 2022-12-12

**Authors:** Xianheng Ouyang, Anliang Chen, Yan Li, Xiaoxiao Han, Haiping Lin

**Affiliations:** 1 School of Forestry and Biotechnology, Zhejiang A&F University, Hangzhou 311300, China; 2 Collaborative Innovation Center of Sustainable Forestry, Nanjing Forestry University, Nanjing 210037, China; 3 College of Plant Protection, Northwest A&F University, Xianyang 712100, China

**Keywords:** pine wilt disease, climate change, MaxEnt model, climate factors, the potential distribution

## Abstract

**Simple Summary:**

Pine forests have been hugely damaged by pine wilt disease (PWD). Climate change may affect the geographic distribution of PWD. Based on 646 PWD infestation sites and seven climate variables, the current and potential geographic distribution of PWD was predicted by using the maximum entropy (MaxEnt) model, which can provide a scientific basis for the prevention and control of PWD. This study shows that the fundamental climate variables influencing PWD distribution were rainfall and temperature. Under different climate scenarios in the future, the areas of potential geographic distribution habitats of PWD will increase to varying degrees compared with the area of modern potential geographic distribution habitats, and the centroid of suitable areas of PWD will move to the northeast.

**Abstract:**

The primary culprits of pine wilt disease (PWD), an epidemic forest disease that significantly endangers the human environment and the world’s forest resources, are pinewood nematodes (PWN, *Bursaphelenchus xylophilus*). The MaxEnt model has been used to predict and analyze the potential geographic spread of PWD in China under the effects of climate change and can serve as a foundation for high-efficiency monitoring, supervision, and prompt prevention and management. In this work, the MaxEnt model’s criteria settings were optimized using data from 646 PWD infestation sites and seven climate variables from the ENMeval data package. It simulated and forecasted how PWD may be distributed under present and future (the 2050s and 2070s) climatic circumstances, and the key climate factors influencing the disease were examined. The area under AUC (area under receiver operating characteristic (ROC) curve) is 0.940 under the parameters, demonstrating the accuracy of the simulation. Under the current climate conditions, the moderately and highly suitable habitats of PWD are distributed in Anhui, Jiangxi, Hubei, Hunan, Guangdong, Guangxi, Sichuan, and other provinces. The outcomes demonstrated that the fundamental climate variables influencing the PWD distribution were rainfall and temperature, specifically including maximum temperature of warmest month, mean temperature of driest quarter, coefficient of variation of precipitation seasonality, and precipitation of wettest quarter. The evaluation outcomes of the MaxEnt model revealed that the total and highly suitable areas of PWD will expand substantially by both 2050 and 2070, and the potential distribution of PWD will have a tendency to spread towards high altitudes and latitudes.

## 1. Introduction

Climate is one of the decisive criteria affecting the distribution of species [1,2]. Recently, urbanization has accelerated, and the natural environment is facing rapid fragmentation [3]. Regarding the 5th assessment report (AR5), the United Nations Intergovernmental Panel on Climate Change (IPCC) stated that the global climate will continue to warm, and the average temperature of the earth will rise between 0.3–4.5 °C towards the end of the 21st century compared with 1986–2005 [4,5]. The seasonal variation of potential evapotranspiration and other climate variables will also change with climate warming [6]. Changes in the structure, function, and stability of ecosystems will directly influence the distribution of species [7,8].

Predicting the distribution of suitable habitats under climate variation has become a major research endeavor [9]. There are a number of models for predicting species’ potential distribution, which include, Climate-matching Tools (CLIMEX), Genetic Algorithm for Rule-set Production (GARP), Ecological Niche Factor Analysis (ENFA), and Maximum Entropy (MaxEnt) [10,11]. MaxEnt has the highest accuracy [12]. It uses a species niche distribution prediction methodology that simulates the distribution probability of species based on known sites currently occupied by species combined with ecological changes in the target distribution area [13]. Its advantage is that the accuracy of its results is high even if the species distribution information is incomplete [14]. It has been broadly implemented in potential planting area estimation, invasive plant distribution area prediction, quarantine pest prediction, and so forth [15,16,17]. Recent research has demonstrated that when the MaxEnt model simulates the potential distribution area of a species, it offers high complexity and is not conducive to model transfer; after the criteria of the MaxEnt model are adjusted by the ENMeval packet, it can better predict potentially suitable areas for species [18].

Pinewood nematode (PWN, *Bursaphelenchus xylophilus*) leads to pine wilt disease (PWD), a worldwide plant disease [19,20]. In Asia, PWD is causing great damage to the environment and may cause major ecological disasters in the future [21]. In China, PWD was first reported in Nanjing in 1982 [22], and since then, it has been reported in Jiangsu, Anhui, Guangdong, and Zhejiang [23]. If a tree is infected with PWD, it can die within a few months [24]. PWD has severely been threatening pine resources in China, causing significant economic losses and ecological consequences to pine forests [25,26]. There were 718 county-level administrative regions in 17 provinces of China damaged by PWD, with 19.5 million dead trees and approximately 1.81 million hectares infected by the end of December 2020 [26]. In the future climate, the distribution of PWD may change. To assess if, in the future, the distribution of PWD may be affected by climate variables, there has been much research regarding the distribution of PWD under climate change. For example, the risk of PWD was predicted by MaxEnt based on a multi-angle and fine-scale study [27]; MaxEnt was used to model the PWD-damaged forest distributions during the period 1982 to 2020 and environmental factors included annual meteorological and human activity factors [26], and three host plants in China were chosen for MaxEnt modeling to simulate the impact of climatic change on PWD [20]. However, unlike earlier studies that employed the MaxEnt model to forecast the suitability of PWD, the current study examined the impact of scenario changes in greenhouse gas emissions under socioeconomic changes and policy interventions on the geographic distribution of species in addition to taking into account the relationship between carbon dioxide concentration and climate [28]. In contrast to representative concentration paths (RCPs), shared socioeconomic pathways (SSPs) can be employed to forecast greenhouse gas emission scenarios under various climate policies in 2100; this approach considers the effects of land use and socioeconomics on the development of regional climate change [14]. The study used historical climatic data from 1970 to 2000 and projected climate data for the 2050s and 2070s based on SSP1-2.6, SSP2-4.5, and SSP5-8.5 to estimate the likely distribution of PWD in China. For the purpose of preventing and controlling PWD, it is vital to have accurate knowledge on its potential distribution and the significant environmental elements that may influence it.

Herein, the improved MaxEnt model was employed for simulating and estimating the prospective distribution of PWD in China based on the distribution data of PWD and pertinent climatic parameters, and ArcGIS software was used to divide the eligible distribution areas. In our study, we aimed to (1) determine the potential habitats of PWD, (2) identify the dominant climate factors influencing its distribution, and (3) predict shifts in its distribution under future climate scenarios to present a foundation for formulating quarantine measures and monitoring management and timely control of PWD.

## 2. Materials and Methods

### 2.1. Incidence Rates for PWD

**Figure 1:**
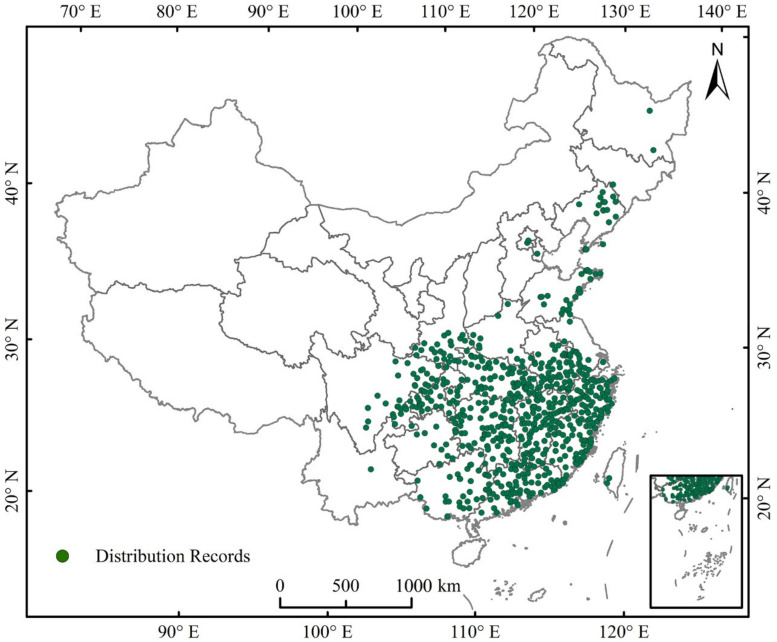
Occurrence records of pine wilt disease in China.

There are many species of pine trees infected with pine wood nematode, and the pine trees infected in different countries or regions are different [21,29]. In China, Japan and South Korea in Asia, the species *Pinus massoniana*, *Pinus densiflora* and *Pinus thunbergii* are susceptible to pine wood nematode disease, which has caused extensive death of pine trees [20]. Tang et al. [20] used MaxEnt to predict the potential distribution of *B. xylophilus* and its hosts, *P. massoniana*, *P. densiflora*, and *P. thunbergii*, under climate change in China. In the study, a MaxEnt model based on the distribution of PWD in China was used to explore and predict the potential distribution of PWD in 2050 and 2070 under two distinct climate change scenarios. Information regarding endemic regions affected by PWD from 1982 to 2021 was acquired through the National Forestry and Grassland Administration (http://www.forestry.gov.cn/main/index.html, accessed on 28 November 2021). Duplicate data points and specimen evidence with unidentified geographic coordinates were eliminated. For available records lacking coordinates, latitude and longitude coordinates for the places named in the data were obtained using Baidu tools (http://api.map.baidu.com/lbsapi/getpoint/index.html, accessed on 28 November 2021). Overall, 646 sites of PWD infestation were selected and saved as a “.csv” file for next utilization (Figure 1).

### 2.2. Selection and Comparison of Climate Variables

A total of 19 environmental factors for the current (1970–2000) and future (2050s and 2070s) were downloaded through WorldClim (http://www.worldclim.org/, accessed on 28 November 2021) using a resolution of 2.5′ (Table S1). Datasets for BCC-CSM2-MR from Coupled Model Intercomparison Project phase 6 were chosen for further climate data, including three scenarios in shared socioeconomic pathways (SSPs), specifically including SSP1-2.6, SSP2-4.5, SSP5-8.5, respectively, representing the low, moderate, high radiative forcing with climate warming [30]. As there is a certain correlation between climate variables, the study performed Spearman correlation analysis on all climate variables in ArcGIS to avoid overfitting in MaxEnt modeling [31]. If the coefficient of correlation for two related variables is more than 0.8, the lower rate of contribution for the variables should be deleted [32]. The seven climate variables finally screened were used in the MaxEnt model for computational analysis (Table 1). The map data were obtained through the website of the Ministry of Natural Resources of the People’s Republic of China (http://www.mnr.gov.cn/, accessed on 28 November 2021).

### 2.3. Optimization of Model Parameters and Model Building

When modeling the possible distribution of a species, the MaxEnt model is easy to overfit, producing unreliable predictions. This could significantly impede its use in disciplines like global change biology and other research [33]. Therefore, the MaxEnt model parameters were modified using the ENMeval data program for this study. The model parameters were optimized with the use of the ENMeval package in R version 3.6.1. [34]. Two regularization multiplier (RM) and feature combination (FC) parameters were controlled, and the complexity of the model for various parameter combinations was examined to determine the combination with the lowest complexity for modeling. The RM parameter was set to 0.5–4 with 0.5-spaced intervals. There are 8 RM parameters in all. Five features were offered by the MaxEnt model for FC: L for linear, Q for quadratic, H for hinge, P for a product, and T for threshold [35]. The six feature combinations L, LQ, H, LQH, LQHP, and LQHPT were chosen. The 48 aforementioned factors were together tested using the ENMeval data program. To evaluate the model’s complexity, the Akaike information criterion correction (AICc) was applied [36,37]. The optimal model parameter combination for MaxEnt model modeling is when the AICC value is the lowest (delta. AICC = 0) [38].

Following, 75% of the samples were selected as the training subset after 646 PWD sites were gathered and input into the MaxEnt model software, while the remaining 25% were used for the model’s verification. The operation was repeated ten times for modeling, with 10,000 being the maximum number of iterations. For evaluating the significance of climate factors restricting the possible geographical distribution of PWD in China, the contribution rate generated by the MaxEnt model and jackknife test was used. Model validity was assessed using the area under the receiver operating characteristic curve (AUC). The value of AUC was 0–1, whereby as AUC approached 1, modeling evaluation outcome authenticity increased [18]. Documents containing the prediction results of PWD were reclassified using ArcGIS 10.5. Jenks’s natural breaks were used [2]. The suitable area for PWD can be divided into four levels: highly suitable (range = 0.50 to 1.00), moderately suitable (range = 0.30 to 0.50), poor (range = 0.10 to 0.30), and unsuitable habitat (range < 0.10). The area of each suitable habitat zone was measured. After 0.14 was set as the threshold, a suitable grade distribution map of PWD was transformed into binary format. Based on the binary map of PWD distribution under the current and future climate change scenarios considered, the COGravity function in the SDMTools package of R language was used to calculate the centroid position of the considerably proper area of PWD disease under current and future climate change. The changes in the centroid of the considerable suitable area of PWD under different climate strategies were compared.

### 2.4. Assessment of Multivariate Environmental Similarity Surface

We used the multivariate environmental similarity surface (MESS) for analyzing levels of ecosystem-based variability of PWD for regions-of-spread in the future. Referencing level for bioclimatic factors was ascertained using MESS analysis. The similarities among bioclimatic factors under various climatic circumstances while pre-determined points for bioclimatic factors within referencing level (similarity, S) were calculated. As the S-value was positive, any reduction in such a reading led to more distinct climatic variations; no variation existed since the S reading was 100. The value of S for at least one bioclimatic factor was beyond the reference range because it was negative. The current state of the climate was favorable [39]. The “Density. Tools. Novel” tool found in the MaxEnt.jar file’s command window was used to make the prediction.

## 3. Results

### 3.1. Model Optimization and Accuracy and Evaluation

**Figure 2:**
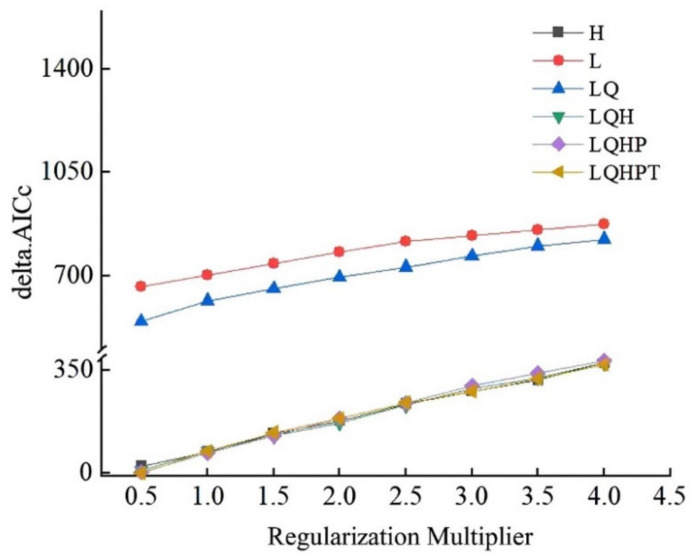
Evaluation metrics of MaxEnt model generated by ENMeval.

Based on 646 distribution sites and seven PWD-specific climate variables, the MaxEnt niche model was utilized to forecast the possible geographic distribution of PWD in China. The MaxEnt model’s default settings were RM = 1, FC = LQHPT. The MaxEnt parameter values were optimized using the ENMeval package. The model’s AICc value was the lowest delta.AICc = 0 when RM = 0.5 and FC = LQHPT (Figure 2). Based on the Akaike information criterion correction, the model manifested the lowest complexity under this parameter. Therefore, FC = LQHPT and RM = 0.5 were set as the optimal model parameters. The optimized parameters were used to remodel and simulate the area suitable for PWD in China. The training data for AUC was 0.940, indicating that the estimation outcomes of the model of MaxEnt were accurate for PWD (Figure S1).

### 3.2. Main Climate Variables Affecting Distribution of Pine Wilt Disease

The contribution rate of climate variables to the adaptability of PWD in China is shown (Table 1). The higher the contribution rate to the probability of the PWD occurrence was rainfall of wettest quarter (bio16, 45.7%), followed by the maximum temperature of the warmest month (bio5, 27.5%) and rainfall in driest month (bio14, 16.1%). The cumulative contribution rate was 89.3%. It shows that these factors had the greatest influence on predicting the probability of PWD. The relative importance of each variable for predicting the probability of species existence was obtained based on the jackknife method (Figure S2). By employing a single climatic factor, the three climatic factors that had the most significant impact on regularized training were rainfall in driest month (bio14), rainfall of wettest quarter (bio16), and mean temperature of driest quarter (bio9) when only a single climatic factor was used. In conclusion, temperature and rainfall were the predominant climatic factors influencing the probable geographic dispersion of PWD. The maximum temperature of the warmest month (bio5), the mean temperature of the driest quarter (bio9), the amount of rainfall during the driest month (bio14), and the amount of rainfall in the wettest quarter (bio16) were used to create a logistic curve within MaxEnt that is suitable for just one climate factor (Figure S3). The suitable distribution grade is high, and the proper range was the probability of existence > 0.5. The proper range of the maximum temperature of warmest month (bio5) was 31–33 °C, that of mean temperature of driest quarter (bio9) was 5.1–20 °C, that of rainfall in driest month (bio14) was 21–64, and that of rainfall of wettest quarter (bio16) was 480–900 mm (Figure S3).

### 3.3. Current Potential Distribution

**Figure 3:**
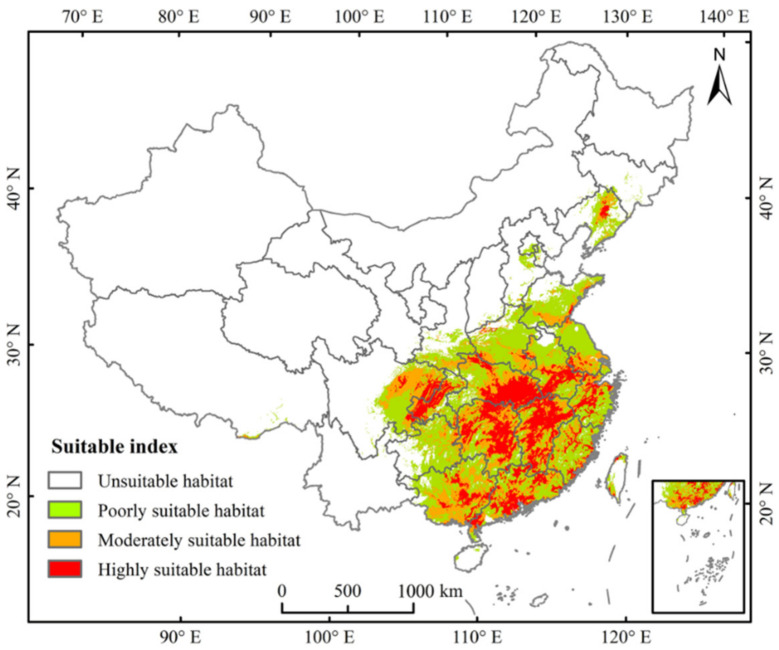
Potential current and suitable habitat for pine wilt disease in China.

The potential distribution of PWD was mainly in central and southeast China under the present climate scenario (Figure 3). We calculated the areas of suitable habitat in each province (Table 2). The total suitable area for PWD was 197.35 × 10^4^ km^2^, the area of highly suitable habitat was 44.11 × 10^4^ km^2^, that of moderately suitable habitat was 55.36 × 10^4^ km^2^, and that of poorly suitable habitat was 97.88 × 10^4^ km^2^ (Table 3). Hunan, Jiangxi, Hubei, Guangdong, Anhui, Chongqing, and Guangxi possess comparatively large areas of remarkably suitable habitat. The area of highly suitable habitat in Jiangxi is 7.84 × 10^4^ km^2^, the best ranking in China. Guangxi, Hunan, Sichuan, Jiangxi, and Hubei have moderate-optimal environment regions compared with other provinces. No suitable PWD regions-of-spread were found within Heilong Jiang, Ningxia, Qinghai, Macao, and Xinjiang.

### 3.4. Potentially Suitable Climatic Distributions in the Future

**Figure 4:**
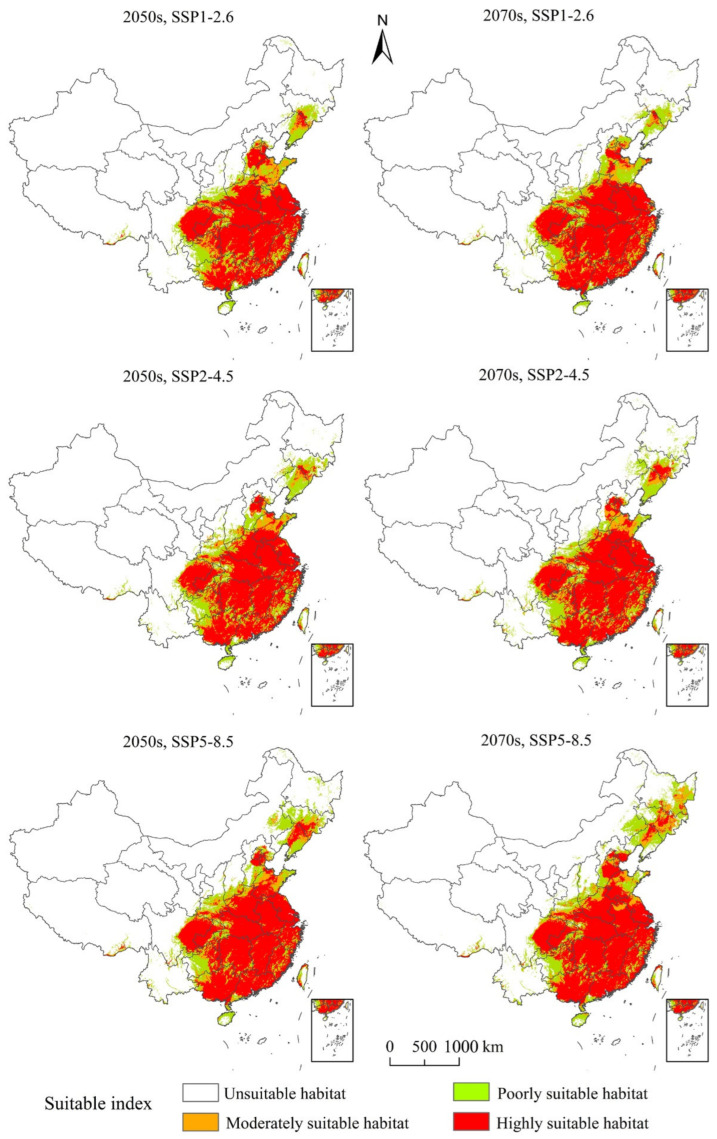
The potentially suitable distribution for pine wilt disease under various further climate change strategies in China.

Under SSP1-2.6, SSP2-4.5, and SSP5-8.5 for the 2050s and 2070s, evaluations of potentially suitable distributions of PWD in the future were illustrated (Table 3). The main distributions were in the south, east, and central China (Figure 4). It can be seen from Table 3, under SSP1-2.6, the total suitable area for PWD was 240.36 × 10^4^ km^2^, 21.79% more than the present distribution. The highly suitable area improved by almost three times its current amount, whereas the moderately suitable and poor areas decreased by 36.36% and 44.63%, respectively, in 2050. Under SSP2-4.5, the total suitable area for PWD was 246.99 × 10^4^ km^2^, an increase of 25.15% in comparison to the current distribution area. The highly suitable area almost tripled, whereas the moderately suitable and poor areas decreased by 39.04% and 36.77%, respectively, in 2050. Under SSP5–8.5, the total suitable area of PWD was 267.23 × 10^4^ km^2^, an increase of 35.41% in comparison to the present distribution area. The highly suitable area increased by almost four times, and the moderately suitable and lowly suitable areas decreased by 40.14% and 37.22%, respectively, in 2050. Under SSP1-2.6, in 2070, the total suitable area of PWD was 243.69 × 10^4^ km^2^, an increase of 23.48% in comparison to the present distribution area. The highly suitable area almost tripled, and the moderately suitable and poor areas decreased by 44.09% and 42.04%, respectively, in 2070. Under SSP2-4.5, the total suitable area of PWD was 251.97 × 10^4^ km^2^, an increase of 27.68% in comparison to the present distribution area. The highly suitable area almost tripled, and the moderately suitable and poor areas decreased by 38.75% and 38.01%, respectively, in 2070. Under SSP5-8.5, the overall proper area of PWD was 286.29 × 10^4^ km^2^, an increase of 45.07% in comparison to the present distribution area, the highly suitable area almost quadrupled, and the moderately suitable and poor areas decreased by 29.12% and 24.48%, respectively, in 2070.

**Figure 5:**
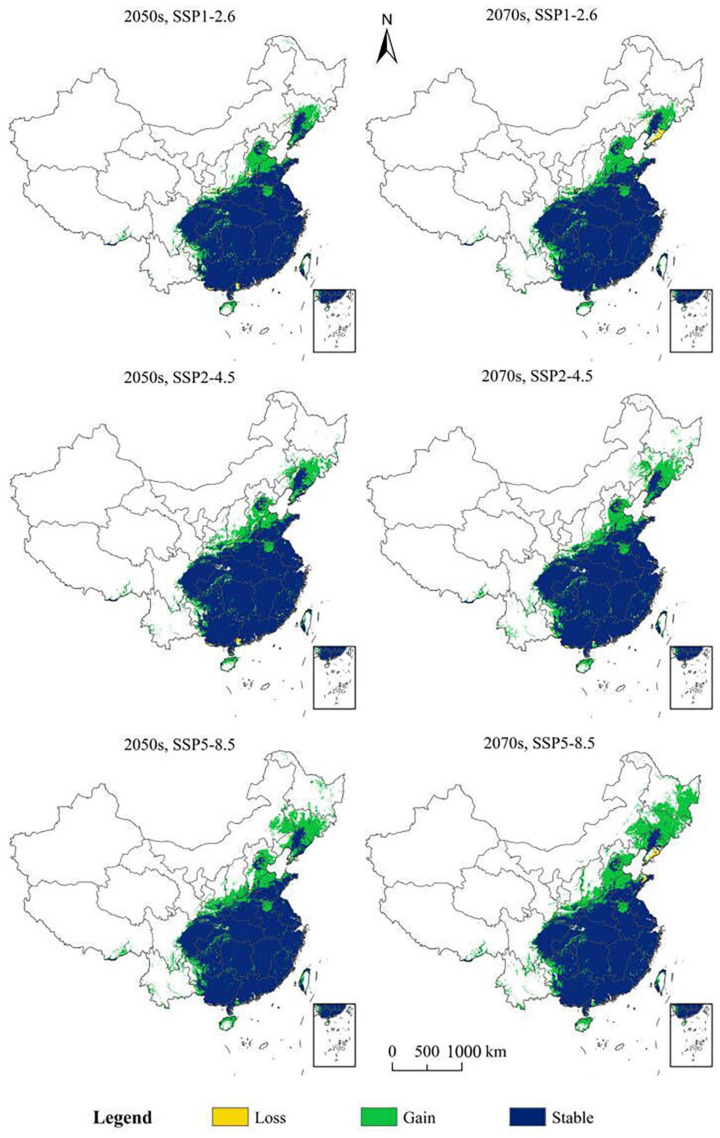
Changes in potentially suitable pine wilt disease habitat under various climate change scenarios in China.

By the 2050s, the total suitable area will increase by 44.01 × 10^4^ m^2^ (SSP1-2.6), 50.72 × 10^4^ m^2^ (SSP2-4.5), and 69.92 × 10^4^ m^2^ (SSP5-8.5) (Table 4). The greatest increases occurred under SSP5-8.5, and these were mainly distributed in parts of Hebei, Liaoning, Shanxi, Shaanxi, Hainan, Hunan, and other regions (Figure 5). By the 2070s, the suitable areas increased by 48.17 × 10^4^ m^2^ (SSP1-2.6), 55.24 × 10^4^ m^2^ (SSP2-4.5), and 90.48 × 10^4^ m^2^ (SSP5-8.5) (Table 4).

### 3.5. Highly Suitable Area Centroid Distributional Shifts under Climate Change for Pine Wilt Disease

**Figure 6:**
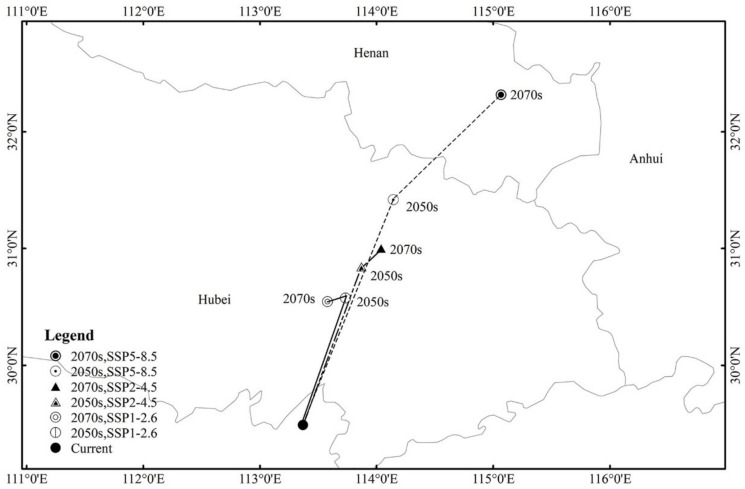
Highly suitable area centroid distributional shifts during climate change in pine wilt disease.

The current centroid of highly suitable habitat for PWD is 113.37 E, 29.49 N. The centroid of highly suitable habitat under SSP1-2.6 moved to 113.74 E, 30.58 N in 2050 and 113.59 E, 30.54 N in 2070. The centroid of highly suitable habitat under SSP2-4.5 shifted to 113.87 E, 30.84 N in 2050 and 114.04 E, 30.99 N in 2070. The centroid of the highly suitable habitat under SSP5-8.5 moved to 114.15 E, 31.41 N in 2050 and 115.06 E, 32.33 N in 2070. In general, the highly suitable area for PWD is shifting to the northeast under future climate change scenarios. The highly suitable area centroid will shift towards Hubei and Henan in the future (Figure 6).

### 3.6. Analysis of the Multivariate Environmental Similarity Surface (MESS) of Potential Area of Distribution for Pine Wilt Disease under Further Climate Change Strategies

**Figure 7:**
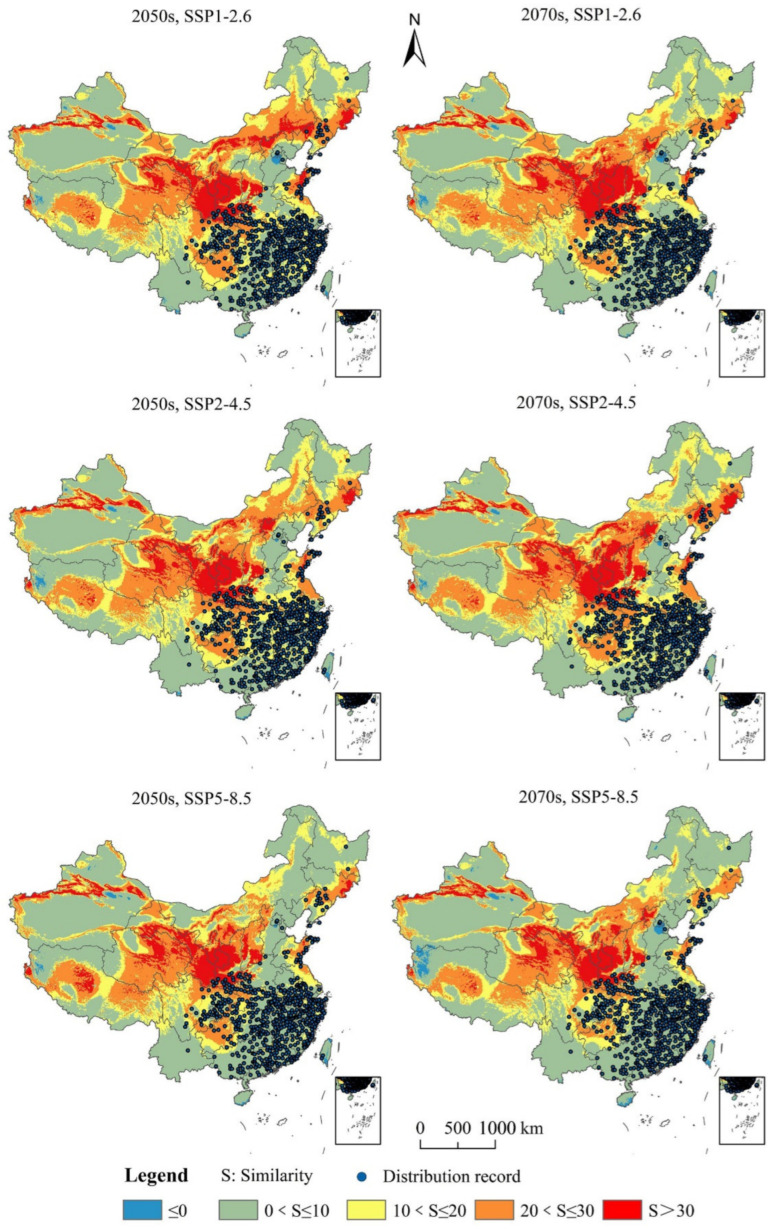
Assessment of the multivariate environmental similarity surface (MESS) of potential pine wilt disease distribution under future climate change scenarios.

Under the future climate scenarios, the distribution of the climate anomaly area (S ≤ 0) in the full prospective distribution area was low (Figure 7). In comparison to the potential distribution region under identical climate scenarios, no suitable PWD habitat was found in the climate anomaly area. According to SSP1-2.6, SSP2-4.5, and SSP5-8.5 in the 2050s and 2070s, respectively, the mean similarity values of the 646 unprecedented effective distributional locations for PWD were 7.73, 6.08, 9.13, 9.15, 5.74, and 5.12. This showed that while the degree of abnormality was minimal in the other five scenarios, it was higher in the 2070s, SSP5-8.5 climate scenario.

## 4. Discussion

Herein, the distribution of PWD was estimated with the MaxEnt model. Environmental factors and known PWN infestation sites were combined in modeling the current and future potential distribution of the species [40]. By examining model complexity under various parameter settings, the parameters with the lowest level of complexity were selected to predict the possible distribution areas of PWD disease [34]. The model’s estimation outputs demonstrated remarkable validity and feasibility, as indicated by the average value of training AUC equal to 0.940.

The study demonstrates that the key meteorological variables influencing PWD distribution were temperature and rainfall. The two most important variables are the mean temperature of the driest quarter (bio9) and the maximum temperature of the warmest month (bio5). *B. xylophilus* and its host, *Monochamus alternatus*, can thrive in a favorable climate; however, the maximum temperature in the warmest month had an impact on the dispersal of adult *M. alternates*, the vector of pine wood nematodes [41]. The maximum temperature of the warmest month ranged from 31–33 °C in the study. Pine trees grown in hot and dry conditions are more susceptible to PWD and the risk of PWD increases when the temperature exceeds 20 °C in summer [42,43,44]. The population density of *B. xylophilus* was significantly increased by high temperature. Naoko et al. [45] studied pine forests which PWD had seriously damaged in the warm temperature zone of Japan. The study shows that rainfall in the driest month (bio14) and rainfall of the wettest quarter (bio16) had a significant influence on PWD distribution in China. The water stress experienced by the host trees is also the crucial climatic factors affecting the PWD distribution; severe drought may cause greater hazards to host plants than PWN, therefore, pine species affected by water scarcity are easy to outbreak PWD [46]. Menéndez-Gutiérrez et al. [47] studied rainfall as a crucial variable affecting damage caused by PWD, reporting considerable pine death during a summer drought. Currently, researchers agree that conditions of high temperature and drought are conducive to the occurrence of PWD [20,48].

Under the six greenhouse gas emission scenarios, the potential distribution area of PWD predicted by MaxEnt will shift to the northeast, and the potentially suitable area will expand considerably. Numerous animals’ living conditions have changed recently as a result of the intensification of global warming. Climate is the key factor influencing the distribution area of species. Pest distribution will be significantly impacted by climate change, and global warming will be the main trend of climate change in the coming decades [49]. According to published studies, as climate change intensifies, the suitable PWD distribution area in China would nearly double by 2100 [50]. According to the study, centroid distributional shifts in highly appropriate areas will typically expand to high latitudes and altitudes. The center of distribution of the *P. massoniana* distribution area was similar to that of *B. xylophilus* under RCP2.6 condition, and to prepare for future climate change, the center of distribution will shift to the northeast [20]. Similar niche demands show their identical response to low representative concentration pathways [51,52]. The identical distribution range and shift in the center of distribution suggests that the host plants will face a continuous disease risk under the scenario. According to MaxEnt, the PWD outbreak in Asia will often spread to the north and higher elevations [53]. The results of the association between *B. xylophilus* and temperature showed that lower temperatures can impede its expansion by influencing the activity range and reproduction of *M. alternatus*; global warming may lead to the activity of the nematode vector *B. xylophilus* that ameliorates damages caused through PWD [54]. Predictions of the potential distribution of PWD need to consider host plants, topography, soil, and human activities. Abiotic and biotic factors and species migration affect the distribution of species during their long-term evolution, and the range of species is different in different historical periods [55]. The period of the climatic elements considered in this study, from 1970 to 2000, leaves out more than two decades of climate data, which could cause some errors in the forecast of places that would be ideal for PWD. In the future study, missing data will be supplemented for predicting the potential distribution of PWD in China based on host plants and more environmental variables to make the prediction results more accurate and reliable.

If effective prevention and control of PWD are not urgently realized, this disease may soon spread across a larger area in China, causing a large number of pine tree deaths every year, amounting to an ecological disaster [56,57]. The techniques adopted to control PWD in China mainly include disease quarantine and epidemic situation monitoring, removing diseased woods that were dead and sick in infected regions, and vector insect control [58]. Further, there is an active disease prevention measure, namely, trunk injection. Trunk injection involves injecting effective components into the tree which are distributed by its transpiration [59]. In order to reach the xylem during a trunk injection, a hole must be drilled through the tree’s bark. Alternatively, a needle may be inserted into the trunk to administer the prepared pesticide. Pesticides may be applied in either a completely concentrated or diluted form, and after being applied, the substance is transported via the xylem and drawn up to the leaves by transpiration [60]. It benefits from a precise application, high control effectiveness, and environmental friendliness [61,62,63]. The application of trunk injection with emamectin benzoate has been widely promoted in the southeast provinces of China [64]. Emamectin benzoate is a semi-synthetic second-generation avermectin-derived insecticide revealed to possess the most potent nematocidal activity against *B. xylophilus* among different chemical compounds [65]. Hence, it could be regarded as a promising alternative as a preventive trunk injection versus PWD.

## 5. Conclusions

According to the actual distribution data of PWD and current (1970 to 2000) and future (2050s and 2070s) climate information, the MaxEnt model was employed for evaluating the potential distribution of PWD in China. The prediction outcome demonstrated that moderately and highly suitable habitats are primarily spread in Anhui, Jiangxi, Hubei, Hunan, Guangdong, Guangxi, and Sichuan under the current climatic and environmental conditions. The entire and highly appropriate habitat area will dramatically grow and spread to the northeast between 2050 and 2070. Temperature and rainfall are the primary climatic elements affecting the possible geographic dispersion of PWD. It is possible to establish a foundation for containment, supervision, and effective prevention and control by forecasting the potential diffusion of PWD.

## Figures and Tables

**Table 1:** Contributions of the climatic factors to the model of MaxEnt.

Climatic Factors	Contribution (%)
Rainfall of wettest quarter (bio16)	45.7
The maximum temperature of warmest month (bio5)	27.5
Rainfall in driest month (bio14)	16.1
Isothermality (bio3)	3.7
Rainfall seasonality (coefficient of variation) (bio15)	3.2
Mean temperature of driest quarter (bio9)	1.9
Temperature seasonality (standard deviation × 100) (bio4)	1.8

**Table 2:** The potential distribution areas for pine wilt disease under current climatic circumstances.

Province Municipality Autonomous Regions	Poorly Suitable Habitat (10^4^ km^2^) 10–30%	Moderately Habitat (10^4^ km^2^) 30–50%	Highly Habitat (10^4^ km^2^) 50–100%	Total Suitable Habitat (10^4^ km^2^)
Beijing	0.41	0.00	0.00	0.41
Tianjin	0.20	0.00	0.01	0.21
Hebei	1.27	0.07	0.01	1.35
Shanxi	0.18	0.01	0.00	0.19
Inner Mongolia	0.04	0.00	0.00	0.04
Liaoning	3.96	1.27	0.55	5.78
Jilin	0.39	0.02	0.01	0.42
Heilongjiang	0.00	0.00	0.00	0.00
Shanghai	0.42	0.10	0.00	0.52
Jiangsu	5.93	2.61	0.57	9.11
Zhejiang	4.61	2.05	2.27	8.93
Anhui	6.16	2.79	3.07	12.02
Fujian	6.15	2.52	1.81	10.48
Jiangxi	2.93	4.41	7.84	15.18
Shandong	7.65	2.25	0.22	10.12
Henan	9.45	3.21	0.50	13.16
Hubei	5.59	4.06	7.04	16.69
Hunan	4.71	6.74	7.60	19.05
Guangdong	5.55	4.18	5.14	14.87
Guangxi	8.77	7.66	2.61	19.04
Hainan	0.18	0.00	0.00	0.18
Chongqing	2.72	1.67	2.62	7.01
Sichuan	6.66	5.95	1.89	14.5
Guizhou	7.98	2.22	0.14	10.25
Yunnan	0.65	0.01	0.00	0.66
Tibet	0.30	0.14	0.01	0.45
Shaanxi	4.30	1.25	0.02	5.57
Gansu	0.04	0.00	0.00	0.04
Qinghai	0.00	0.00	0.00	0.00
Ningxia	0.00	0.00	0.00	0.00
Xinjiang	0.00	0.00	0.00	0.00
Macao	0.00	0.00	0.00	0.00
Taiwan	0.64	0.15	0.18	0.97
Xianggang	0.04	0.02	0.00	0.06
Total (China)	97.88	55.36	44.11	197.35

**Table 3:** Suitable areas for pine wilt disease under various climatic conditions.

Decades Scenarios	Predicted Area/10^4^ km^2^			
	Poorly Suitable Habitat	Moderately Suitable Habitat	Highly Suitable Habitat	Total Suitable Habitat
Current	97.88	55.36	44.11	197.35
2050s, SSP1-2.6	54.2	35.23	150.93	240.36
2050s, SSP2-4.5	61.89	33.75	151.35	246.99
2050s, SSP5-8.5	61.45	33.14	172.64	267.23
2070s, SSP1-2.6	56.73	30.95	156.01	243.69
2070s, SSP2-4.5	60.68	33.91	157.38	251.97
2070s, SSP5-8.5	73.92	39.24	173.13	286.29

**Table 4:** Future changes in suitable habitat area.

Decades, Scenarios	Predicted Area/10^4^ km^2^		
	Loss	Gain	Stable
2050s, SSP1-2.6	1.52	44.01	196.35
2050s, SSP2-4.5	1.56	50.72	196.33
2050s, SSP5-8.5	0.42	69.92	197.39
2070s, SSP1-2.6	2.34	48.17	195.52
2070s, SSP2-4.5	1.11	55.24	196.79
2070s, SSP5-8.5	1.82	90.48	196.01

## Data Availability

The data presented in the study are available in the manuscript.

## Supplementary Materials

The following supporting information can be downloaded at: https://www.mdpi.com/article/10.3390/insects13121147/s1, Figure S1, ROC response curve under the MaxEnt model. Figure S2, Jackknife test of climatic factors for pine wilt disease. Figure S3, Response curves of the main climate factors affecting distribution of pine wilt disease. Table S1, 19 Biocllimatic factors in the research.
