# Supplementary material for: Predicting the Potential Distribution of Pine Wilt Disease in China under Climate Change
Source: Insects. 2022 Dec 12;13(12):1147. doi: 10.3390/insects13121147 (PMC9786912; doi:10.3390/insects13121147)
Supplement: Supplementary file 1 [file insects-13-01147-s001.zip › insects-1957800-supplementary.pdf]

insects

Article

Predicting the Potential Distribution of Pine Wilt Disease in China under Climate Change

Xianheng Ouyang [1], Anliang Chen [1], Yan Li [2], Xiaoxiao Han [3] and Haiping Lin [1,*]

[1] School of Forestry and Biotechnology, Zhejiang A&F University, Hangzhou 311300, China; oyxianheng@163.com (X.O.); anlchen@126.com (A.C.)
[2] Collaborative Innovation Center of Sustainable Forestry, Nanjing Forestry University, Nanjing 210037, China; lyle@njfu.edu.cn
[3] College of Plant Protection, Northwest A&F University, Xianyang 712100, China; hxxlife233@163.com
* Correspondence: hplin@zafu.edu.cn

Table S1. 19 Biocllimatic factors in the research.

Variables	Description	Variables	Description
bio1	Annual mean temperature	bio11	Mean temperature of coldest quarter
bio2	Mean diurnal range (mean of monthly (maximum-minimum temperature))	bio12	Annual rainfall
bio3	Isothermality (bio2/bio7) (×100)	bio13	Rainfall of wettest month
bio4	Temperature seasonality (standard deviation×100)	bio14	Rainfall in driest month
bio5	The maximum temperature of warmest month	bio15	Rainfall seasonality (coefficient of variation)
bio6	The minimum temperature of coldest month	bio16	Rainfall of wettest quarter
bio7	Temperature annual range (bio5-bio6)	bio17	Rainfall of driest quarter
bio8	Mean temperature of wettest quarter	bio18	Rainfall of warmest quarter
bio9	Mean temperature of driest quarter	bio19	Rainfall of coldest quarter
bio10	Mean temperature of warmest quarter		

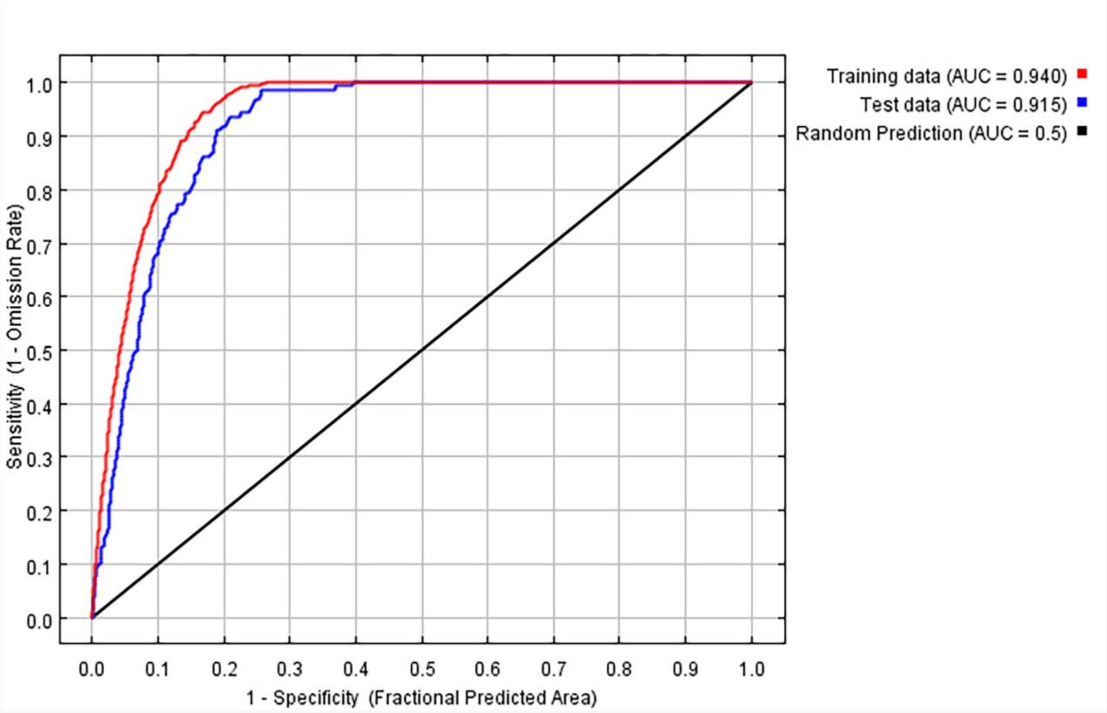

Figure S1. ROC response curve under the MaxEnt model.

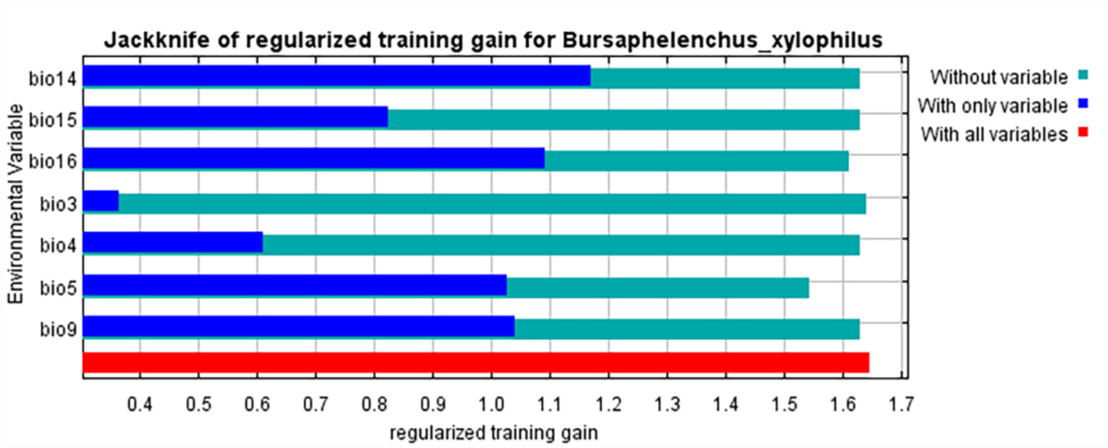

Figure S2. Jackknife test of climatic factors for pine wilt disease.

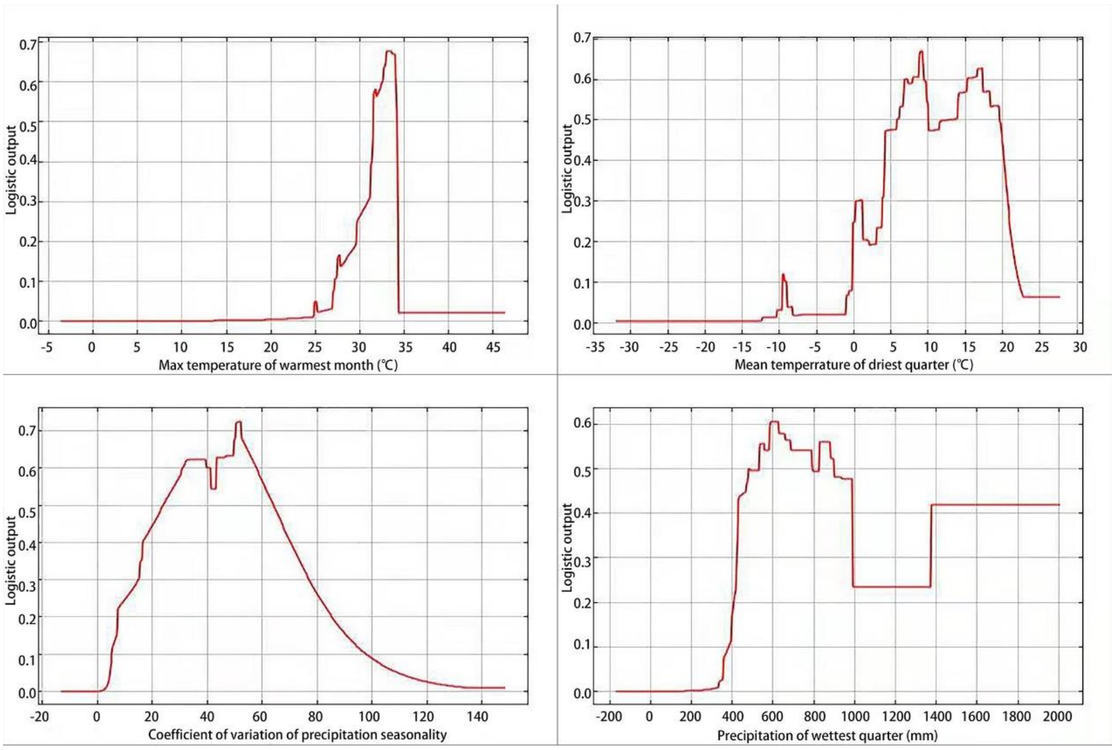

Figure S3. Response curves of the main climate factors affecting distribution of pine wilt disease.
